# Methamphetamine and Parkinson's Disease

**DOI:** 10.1155/2013/308052

**Published:** 2013-02-07

**Authors:** Noelia Granado, Sara Ares-Santos, Rosario Moratalla

**Affiliations:** ^1^Instituto Cajal (CSIC), Avenida Doctor Arce 37, 28002 Madrid, Spain; ^2^CIBERNED, Instituto de Salud Carlos III, Madrid, Spain; ^3^Facultad de Medicina, Universidad Complutense de Madrid, Madrid, Spain

## Abstract

Parkinson's disease (PD) is a neurodegenerative disorder predominantly affecting the elderly. The aetiology of the disease is not known, but age and environmental factors play an important role. Although more than a dozen gene mutations associated with familial forms of Parkinson's disease have been described, fewer than 10% of all cases can be explained by genetic abnormalities. The molecular basis of Parkinson's disease is the loss of dopamine in the basal ganglia (caudate/putamen) due to the degeneration of dopaminergic neurons in the substantia nigra, which leads to the motor impairment characteristic of the disease. Methamphetamine is the second most widely used illicit drug in the world. In rodents, methamphetamine exposure damages dopaminergic neurons in the substantia nigra, resulting in a significant loss of dopamine in the striatum. Biochemical and neuroimaging studies in human methamphetamine users have shown decreased levels of dopamine and dopamine transporter as well as prominent microglial activation in the striatum and other areas of the brain, changes similar to those observed in PD patients. Consistent with these similarities, recent epidemiological studies have shown that methamphetamine users are almost twice as likely as non-users to develop PD, despite the fact that methamphetamine abuse and PD have distinct symptomatic profiles.

## 1. Parkinson's Disease

Parkinson's disease (PD) is the second most common neurodegenerative disorder after Alzheimer's disease, affecting an estimated 7 to 10 million people worldwide. Incidence of the disease increases with age. PD usually affects people over the age of 50, but an estimated 4% of PD cases is diagnosed before the age of 50. Early in the course of the disease, the most obvious symptoms are movement-related. These include shaking, rigidity, slowness of movement, and difficulty with walking and gait. Later, cognitive and behavioral problems may arise, with dementia commonly occurring in the advanced stages of the disease. Other symptoms include sensory, sleep, and emotional problems. PD is caused by degeneration of midbrain dopaminergic neurons that project to the striatum. The loss of striatal dopamine is responsible for the major symptoms of the disease. Although a small proportion of cases can be attributed to known genetic factors, most cases of PD are idiopathic. While the aetiology of dopaminergic neuronal demise is elusive, a combination of genetic susceptibilities, age, and environmental factors seems to play a critical role [1]. Dopamine degeneration process in PD involves abnormal protein handling, oxidative stress, mitochondrial dysfunction, excitotoxicity, apoptotic processes, and microglial activation/neuroinflammation.

## 2. Epidemiology and Pharmacology of Methamphetamine Use

Methamphetamine is an addictive, highly water-soluble CNS (central nervous system) stimulant. It belongs to the group of synthetic drugs chemically related to amphetamine; however, its effects on the CNS are much more pronounced than those of the parent compound. Abuse of these illegal psychostimulants has become an international public health problem, with an estimated 14 to 52 million amphetamine-type stimulant users worldwide, exceeding the total number of cocaine abusers and second only to the number of cannabis abusers [2]. Hydrochloride methamphetamine, known as “meth” or “speed”, can be found in the powder state, compressed into tablets or capsules of 10 to 15 mg, or in a purer crystalline form. 

Methamphetamine is taken by abusers for several desired effects: euphoria and a sense of well-being, increased physical activity and energy, and decreased anxiety. These effects appear immediately after drug consumption and can last for several hours. They may be accompanied by acute adverse effects such as increased blood pressure and heart rate, which may cause irreversible damage to blood vessels in the brain, resulting in cerebrovascular accidents, stroke, and death. Methamphetamine also produces hyperthermia, mydriasis (pupil dilation), flushing, tremors, trismus and bruxism, muscle tension, loss of appetite or anorexia, and loss of pleasure in food intake. 

Methamphetamine is an addictive drug, and abusers may rapidly develop tolerance. The most common symptoms of chronic methamphetamine abuse are temporomandibular joint syndrome, dental erosion, and myofacial pain [3]. Long-term use also produces lack of appetite, weight loss, accelerated aging, nose-bleeding problems, nonhealing wounds, and tooth decay and fracture known as “Meth mouth”. Psychiatric symptoms include anxiety, depression, increased aggression, social isolation, psychosis, mood disturbances, and psychomotor dysfunction. Long periods of high consumption can cause paranoid psychosis. In addition, deficits in attention, working memory, and decision making have been detected in chronic methamphetamine addicts. Withdrawal from methamphetamine can cause irritability, fatigue, impaired social functioning, and intense craving for the drug. There is evidence that the negative neuropsychiatric consequences of methamphetamine abuse are due, at least in part, to drug-induced neuropathological changes in the brain [4].

## 3. Methamphetamine Toxicity in Experimental Animals

### 3.1. Methamphetamine Toxicity in the Striatum

**Figure 1:**
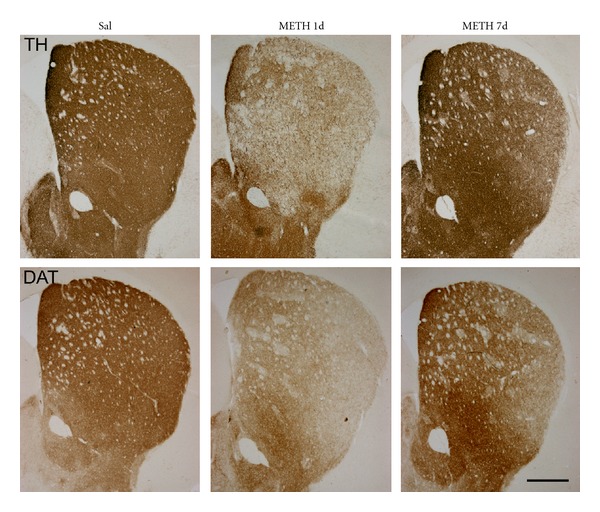
Time-course of TH and DAT fiber lost change after methamphetamine administration. Photomicrographs of striatal sections from mice treated with saline or METH stained for TH and DAT to illustrate the loss (1 day) and the partial recovery (7 days) of dopamine fibers that occur after methamphetamine administration. Animals were killed 1 and 7 days after treatment. Bar indicates 500 *μ*m.

Animal studies have shown that methamphetamine can cause long-term dopamine terminal damage as well as dopamine neuronal body loss. In rodents, repeated administration of methamphetamine causes a decrease in dopaminergic markers such as tyrosine hydroxylase (TH) and dopamine transporter (DAT) (see Figure 1), accompanied by a reduction in TH activity, reduced levels of dopamine (DA) and its metabolites (3,4-dihidroxyphenylacetic, DOPAC, homovanillic acid, HVA), and decreased levels of vesicular monoamine transporter 2 (VMAT2). These effects occur primarily in the striatum (caudate-putamen), but as well in the cortex, thalamus, hypothalamus, and hippocampus [5–10]. Methamphetamine induces neurotoxicity in a dose-dependent manner [11] as do other amphetamine-derivatives like MDMA [12, 13]. Although partial recovery of TH and DAT fibers occurs after methamphetamine administration, methamphetamine-induced neurotoxicity is persistent. In mice, the greatest dopaminergic fiber loss is seen 1 day after methamphetamine administration (Figure 1). Neurotoxic effects persist for more than seven days after methamphetamine exposure [5, 14, 15] and one month after MDMA exposure [13]. Drugs that induce parkinsonian symptoms and TH loss such as MPTP in mice also show a partial recovery with time in nonhuman monkeys and mice [16]. The time courses and degrees of TH and DAT fiber recovery after methamphetamine or after MDMA exposure are similar, suggesting terminal regrowth, as these two proteins are independently regulated (Figure 1). In addition, there is partial recovery of dopamine levels in the striatum [5, 7, 12], strongly suggesting that the regrown terminals are functional. The mechanisms responsible for the partial recovery are not known, but might involve compensatory sprouting and branching as has been reported for regrowth following MPTP-induced damage [17]. Dopamine terminal recovery has also been described in rhesus monkeys and velvet monkeys, although it appears to occur on a slower timescale than in mice: methamphetamine-induced dopaminergic damage persists for more than 12 weeks in velvet monkeys and more than 3 years in rhesus monkeys [11, 18], demonstrating the persistence of methamphetamine-induced brain damage.

Interestingly, striatal TH cells that appear in Parkinsonian brains [19] and in 6-OHDA- and MPTP-denervated animals [20, 21] are also evident after methamphetamine treatment (unpublished observations). These TH neurons only appear in severely dopamine-denervated striatal areas and, therefore, represent evidence in support of the strong denervation that methamphetamine use can cause. 

### 3.2. Methamphetamine Toxicity in the Substantia Nigra

In addition to TH fiber loss, methamphetamine administration produces dopamine cell body loss in the substantia nigra pars compacta (SNpc), as indicated by stereological counts in TH-stained SN sections from mice treated with 3 methamphetamine injections (5 mg/kg) at 3-hour intervals. These counts show 20 to 25% dopaminergic cell loss, measured at different time points after methamphetamine exposure. The observed pattern of TH-stained neuron loss is very similar to the pattern of Nissl-stained neuron loss, indicating that neuronal loss is specific to dopaminergic neurons. Dopamine cell body loss was confirmed via staining with Fluoro-Jade, a general marker of neuronal degeneration that fluoresces after administration of known dopaminergic toxins such as 6-OHDA and MPTP [22]. Fluoro-Jade stains scattered neurons degenerated in the SNpc after methamphetamine treatment. It is possible that the lack of complete recovery of TH fibers in the striatum is related to the loss of dopaminergic neurons in the SNpc [5, 7, 15, 23, 24], resembling what occurs in Parkinson's disease [16]. 

### 3.3. Neurotoxicity Pattern of Methamphetamine

**Figure 2:**
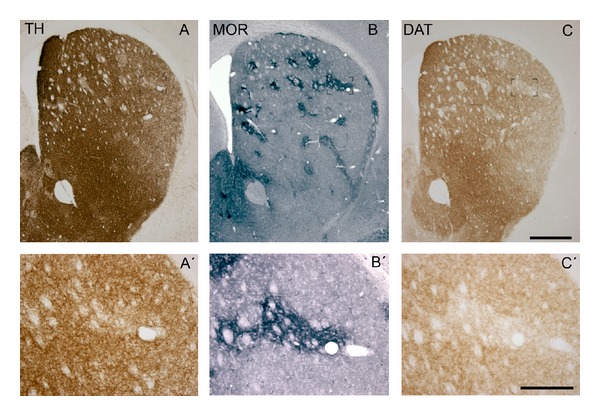
TH- and DAT-ir loss is predominant in striosomes. Serially adjacent sections from a mouse treated with METH stained for TH (A), MOR-1 (B), and DAT (C). Most striatal TH weak patches matched DAT weak patches. These areas corresponded with striosomes as demonstrated by MOR-1 immunostaining. A′–C′ show an example of a striosome at higher magnification. Bar indicates 500 *μ*m (A–C) and 200 *μ*m, (A–C′). Modified from Granado et al. [6].

As in PD, in which the nucleus accumbens is more resistant to dopamine loss than the putamen [25, 26], methamphetamine-induced dopaminergic loss occurs mainly in the nigrostriatal dopaminergic pathway, while the mesolimbic pathway is more resistant [6]. Moreover, the two functional and cytoarchitectonic compartments of the striatum, the striosomes and matrix, have different vulnerabilities to methamphetamine. Striosomes, which are connected with the limbic system and functionally associated with reward-related and emotional behaviours [27, 28], are more vulnerable to methamphetamine-induced dopaminergic terminal loss than the matrix (Figure 2; see also [6]), which is connected to sensorimotor regions of the brain closely associated with motor functions [29]. Similarly, greater striatal damage is observed in the striosomes than the matrix in experimental animals following the administration of other neurotoxins such as MDMA [12], MPTP [30], or quinolinic acid [31]. It is also seen in the early stages of Huntington's disease [32] and following ischemia/reperfusion injury [33, 34]. This pattern of neurotoxicity is inversely correlated with SOD (superoxide dismutase) expression in the striatum, suggesting that striosomes, which have lower levels of SOD expression than the matrix, are more vulnerable because they have less antioxidant capacity [6, 12].

### 3.4. Molecular Mechanisms of Methamphetamine Induced-Neurotoxicity

**Figure 3:**
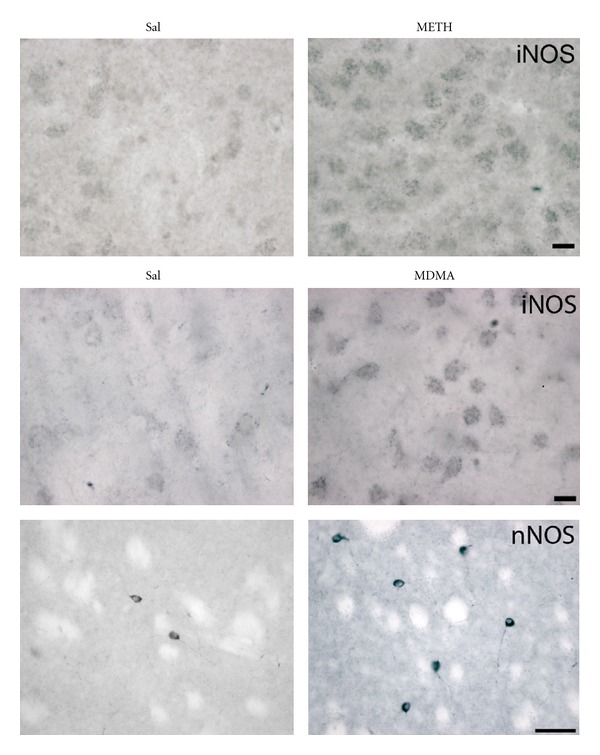
Methamphetamine and MDMA increase the expression of inducible nitric oxide synthase (iNOS) and neuronal nitric oxide synthase (nNOS) in mouse striatum. Photomicrographs of striatal sections of mice treated with saline or methamphetamine (5 mg/kg × 3) or MDMA (20 mg/kg × 3) stained for iNOS and nNOS. Animals were killed 1 day after treatment. Bar indicates 10 *μ*m for iNOS and 50 *μ*m for nNOS. Modified from Granado et al. [13].

Although the exact molecular mechanisms of neuronal body loss are not known, there is evidence to suggest the coexistence of different types of cell death, including apoptosis (indicated by the presence of apoptotic- and AIF-positive-cell bodies) and necrosis (indicated by the morphology of neurons stained with hematoxylin-eosin). Increasing evidence demonstrates that methamphetamine and MDMA induce an increase in lipid peroxidation and DNA oxidation as well as increased levels of oxidative stress markers such as hydroxyl radical producing neurotoxicity [35]. Methamphetamine increases expression of nNOS/iNOS (Figure 3) indicating increased synthesis of neuronal nitric oxide [5, 7, 15], which combines with superoxide radicals to form peroxynitrite, a strong oxidant and a major neurotoxin [36]. Induction of nNOS/iNOS by methamphetamine or MDMA (Figure 3) constitutes part of the mechanism of methamphetamine damage, as selective inhibition or genetic inactivation of nNOS and overexpression of cupper zinc superoxide dismutase (CuZnSOD), an enzyme that catalyzes the dismutationof superoxide into oxygen and hydrogen peroxide, prevent methamphetamine neurotoxicity [23, 37, 38]. Although methamphetamine increases iNOS expression in the striatum (see Figure 3) [5, 6], there is no basis for supposing the involvement of glial nitric oxide in methamphetamine-induced toxicity, but it is interesting to note that mice deficient in iNOS have increased resistance to methamphetamine-induced dopamine neuron damage [39].

**Figure 4:**
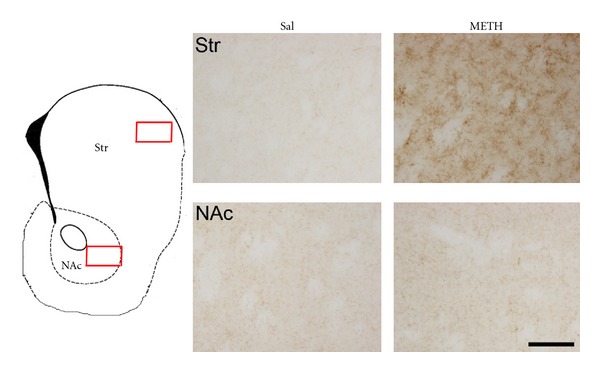
Metamphetamine produces microglial activation in mouse striatum (Str) but not in nucleus accumbens (NAc). Photomicrographs of sections of Str and NAcc of mice treated with saline or metahmphetamine (5 mg/kg × 3) stained for Mac-1. Animals were killed 1 day after methamphetamine treatment for Mac-1. Bar indicates 100 *μ*m.

The neurotoxic effects of methamphetamine on the dopaminergic system are accompanied by activation of astroglia and microglia in the same areas [5, 7, 14, 15, 39–41] being strongest in the striatum (Figure 4), the area with biggest toxicity. Glial cells are not activated in the nucleus accumbens, which is not much damaged (Figure 4). In mice, glial activation in striatum and in substantia nigra occurs shortly after methamphetamine administration, as indicated by a significant increase in Mac-1 (a marker of reactive microglia) 24 hours after methamphetamine exposure (Figure 4), and prominent increases in GFAP (a marker of reactive gliosis in response to injury) occur 3–7 days after treatment [5, 15]. The extent of these glial reactions correlates with the observed severity of neurotoxicity [5, 7, 15].

The dopaminergic system is also involved in this toxicity, as demonstrated in various mutant mice in which inactivation of DAT [42], dopamine D1 receptors [5] or D2 receptors [7] affords a significant protection against methamphetamine toxicity [43]. Administration of THC prevents dopaminergic toxicity after MDMA, a similar amphetamine derivative to methamphetamine, by CB1 receptor stimulation which is present in striatal medium spiny neurons [44]. All these receptors are involved in different aspects of learning processes [45–47] that became affected by the chronic use of methamphetamine or MDMA [3, 4, 48, 49].

## 4. Clinical Toxicology of Methamphetamine 

In light of the methamphetamine-induced dopaminergic neurotoxicity and dopamine loss observed in experimental animals, it has been speculated for years that methamphetamine use may predispose consumers to developing neurodegenerative disorders like Parkinson's disease [4, 50, 51]. However, there were no clinical studies proving this hypothesis until recent epidemiological and neuroimaging reports. Neuroimaging studies in humans have started to elucidate the relationship between methamphetamine-abuse and toxicity and susceptibility to neurodegenerative disorders [3, 52].

### 4.1. Neuroimaging Studies in Human Abusers: PET and MRI Results

**Figure 5:**
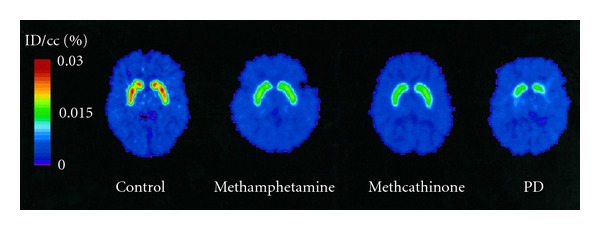
Reduced DAT function in methamphetamine users. PET images showing accumulation of (11C) WIN-35 428 in the striatum in a control subject, an abstinent methamphetamine subject, an abstinent methcathinone subject, and a PD patient 70–90 min after injection of (11C) WIN-35 428. Taken from McCann et al. [53].

Methamphetamine use causes significant long-term dopaminergic neurotoxicity and neurodegeneration in human abusers, and these effects persist long after cessation of drug use. Similar to what has been seen in animal studies, striatal dopamine levels are reduced by ~50% in the brains of human chronic methamphetamine users [52]. Also consistent with animal studies, positron emission tomography (PET) of methamphetamine abusers revealed persistent and significant decreases of 20–30% in dopamine transporter (DAT) in the caudate nucleus and putamen in comparison to control subjects (see Figure 5). This reduction is evident even in abusers who had been detoxified for at least 11 months. Other studies in abstinent former methamphetamine users have demonstrated reductions in DAT binding densities in the striatum as long as 3 years after methamphetamine withdrawal [53]. This DAT reduction in former addicts has been associated with motor slowing and memory impairment [54–56]. 

PET studies also found lower densities of serotonin transporter and vesicular monoamine transporter (VMAT2) across striatal subregions, midbrain, and hypothalamus of methamphetamine users [57, 58]. In addition, methamphetamine users exhibited increased levels of the lipid peroxidation products 4-hydroxynonenal and malondialdehyde in the caudate and frontal cortex [59] and increased levels of the antioxidant compounds CuZnSOD and glutathione in the caudate nucleus [60]. 

PET studies have revealed that human methamphetamine abusers show prominent microglial activation in the midbrain, striatum, thalamus, and orbitofrontal and insular cortices similar to that observed in experimental animals after methamphetamine treatment, with the magnitude of activation inversely correlated to duration of methamphetamine abstinence [61]. Chronic methamphetamine users who died of drug intoxication showed a significant increase in the number of microglial cells in the striatum examined by immunohistochemistry [62]. Intriguingly, several studies have shown that PD patients have more reactive glial cells than do patients without the disease, indicating a possible link between methamphetamine abuse and predisposition to development of PD [63, 64].

Magnetic resonance imaging (MRI) studies demonstrate enlarged striatal volumes in adults who recently abstained from methamphetamine, those with greater cumulative methamphetamine use or longer duration of use, had smaller striatal structures that indicate that the pattern of brain alterations associated with chronic methamphetamine abuse in humans is consistent with cognitive impairment [57]. Moreover, individuals with smaller striatal volumes also performed more poorly on several tests that involved executive function (verbal fluency) and fine motor function (nondominant grooved pegboard). These findings suggest that although methamphetamine use may be associated initially with enlargement of the striatal structures, probably as a compensatory (inflammatory) response, and preserved cognitive function, the volumes of the striatum ultimately decrease with greater methamphetamine usage, accompanied by cognitive impairment. Methamphetamine abusers have increased brain glucose metabolism in the limbic and orbitofrontal regions but relative decreases in the striatum (greater decrease in caudate than in putamen) and in the thalamus [57]. Reductions in DAT levels in the striatum and orbitofrontal and dorsolateral prefrontal cortex have been correlated with the duration of methamphetamine use and the severity of psychiatric symptoms such as anxiety, depression, and psychosis [57, 65]. Furthermore, methamphetamine abusers show severe gray matter decreases in cingulate, limbic, and paralimbic cortices [66] and enlarged striatal volumes [57]. In addition, MR spectroscopy shows reduced concentrations of a marker of neuronal integrity, N-acetylaspartate and total creatine in the basal ganglia [57]. All these findings indicate that methamphetamine abuse is associated with persistent physiologic changes in the human brain, similar to those seen in experimental animals, and that these changes are accompanied by motor and cognitive deficits [67].

### 4.2. Motor and Behavioural Deficits in Methamphetamine Abusers

Although the dopaminergic damage seen in methamphetamine abuse and PD is similar, the symptomatology is largely different. None of the symptoms of methamphetamine abuse is similar to the clinical features of Parkinson's disease; thus, there is no symptomatic reason to expect that PD will arise due to drug-induced dysfunction in the dopaminergic system [68]. Although motor deficits have been reported in chronic methamphetamine abusers, these deficits do not typically involve gross movements, as in PD, but rather affect fine motor dexterity, for example, placing pegs in a pegboard [4, 69]. A plausible explanation for this lack of immediate parkinsonian symptomatology was given by Moszczynska et al., [68] who found that in methamphetamine users, mean dopamine levels were more reduced in the caudate (−61%) than in the putamen (−50%), a pattern opposite to that seen in Parkinson's disease [70, 71]. Some methamphetamine users had dopamine levels within the parkinsonian range (up to 97% dopamine loss) in the caudate but not in the putamen. As the putamen and caudate subserve aspects of motor and cognitive function, respectively, the authors suggested that methamphetamine users were not parkinsonian because dopamine levels are not sufficiently decreased in the motor component of the striatum. However, the near-total reduction of dopamine in the caudate could explain reports of cognitive disturbances, sometimes disabling, in some drug users [69]. 

### 4.3. Increased Risk of Parkinson's Disease in Methamphetamine Abusers

Recent publications examining the connection between methamphetamine abuse and development of PD indicate a correlation between drug use and later development of the disease. Callaghan et al. [72] reported an increase in incidence of PD in methamphetamine users in an epidemiological investigation based on data from California statewide hospital discharge records. They identified 1,863 methamphetamine users, 9,315 patients hospitalized for appendicitis as a nondrug control group, and 1,720 cocaine users as a drug control group. All subjects were aged at least 50 years, had been hospitalized in California between 1990 and 2000, and had been followed for up to 10 years after discharge. The methamphetamine user group showed an elevated incidence of PD, with a 165% higher risk for development of PD than the patients from the control group. These results have been reproduced later by the same group [73], using a larger- and more-age-diverse group of patients (40,000 people hospitalized for methamphetamine versus 200,000 for appendicitis and 35,000 for cocaine) and a 16-year follow-up period. These two studies are the first to link methamphetamine abuse in young adulthood with development of PD in middle age or later, strongly supporting that methamphetamine use increases the risk for developing PD. 

## 5. Conclusions

In experimental animals, exposure to methamphetamine damages dopaminergic fibres in the striatum and their cell bodies in the substantia nigra, echoing the degeneration pattern observed in human patients with PD. Selective damage to dopaminergic terminals in the striatum has also been observed in human methamphetamine users, although there is no evidence so far that methamphetamine damages dopaminergic cell bodies in the human SNpc. Given these results, it is reasonable to think that methamphetamine use may predispose consumers to future development of PD. This hypothesis has been supported by recent epidemiological work indicating that methamphetamine users have an increased risk of developing PD. This is consistent with the persistent neurotoxic effects of methamphetamine in experimental animals and suggests that methamphetamine use may also produce irreversible loss of dopaminergic neurons in the SNpc of human abusers.

PD is a progressive disorder with a presymptomatic interval; that is, there is a period during which the pathologic process has begun, but the motor signs required for clinical diagnosis are absent [51]. Methamphetamine can reduce dopamine levels in the nigrostriatal system significantly before motor symptoms become evident, which may explain why methamphetamine abusers do not display parkinsonism in the early stages of drug consumption. Given the large number of methamphetamine users worldwide, the relationship between methamphetamine intake and PD could become a vast public health problem in the future. 

Further investigation is needed to elucidate the causes and mechanisms of methamphetamine-induced damage. This information will identify mechanisms that might also be involved in pathology of PD and highlight potential new therapeutic strategies for prevention or reduction of dopaminergic neurodegeneration.
